# Production of Banana Fiber Yarns for Technical Textile Reinforced Composites

**DOI:** 10.3390/ma9050370

**Published:** 2016-05-13

**Authors:** Zaida Ortega, Moisés Morón, Mario D. Monzón, Pere Badalló, Rubén Paz

**Affiliations:** 1 Departamento de Ingeniería de Procesos, Universidad de Las Palmas de Gran Canaria, Las Palmas de Gran Canaria 35017, Spain; 2 Acondicionamiento Tarrasense Asociación—LEITAT, Terrassa 08225, Spain; mmoron@leitat.org (M.M.); pbadallo@leitat.org (P.B.); 3 Departamento de Ingeniería Mecánica, Universidad de Las Palmas de Gran Canaria, Las Palmas de Gran Canaria 35017, Spain; mario.monzon@ulpgc.es (M.D.M.); ruben.paz@ulpgc.es (R.P.)

**Keywords:** banana fibers, enzymatic treatment, yarn, composites, reinforcement, sustainability

## Abstract

Natural fibers have been used as an alternative to synthetic ones for their greener character; banana fibers have the advantage of coming from an agricultural residue. Fibers have been extracted by mechanical means from banana tree pseudostems, as a strategy to valorize banana crops residues. To increase the mechanical properties of the composite, technical textiles can be used as reinforcement, instead of short fibers. To do so, fibers must be spun and woven. The aim of this paper is to show the viability of using banana fibers to obtain a yarn suitable to be woven, after an enzymatic treatment, which is more environmentally friendly. Extracted long fibers are cut to 50 mm length and then immersed into an enzymatic bath for their refining. Conditions of enzymatic treatment have been optimized to produce a textile grade of banana fibers, which have then been characterized. The optimum treating conditions were found with the use of Biopectinase K (100% related to fiber weight) at 45 °C, pH 4.5 for 6 h, with bath renewal after three hours. The first spinning trials show that these fibers are suitable to be used for the production of yarns. The next step is the weaving process to obtain a technical fabric for composites production.

## 1. Introduction

The rising interest in natural fibers in the composites field is undeniable, mainly due to sustainability, but also because of their good mechanical properties and low cost. The differences observed among different natural fibers are due to their chemical composition, origin, climate conditions, *etc*. On average, vegetable fibers are made of 60%–70% of cellulose, 10%–20% of hemicellulose, 5%–15% of lignin and up to 2% of pectin and waxes [1].

Banana fiber is obtained from the superimposed leaves forming the pseudostem of the plant, which currently has no use, apart from a low percentage dedicated to cattle feed. It belongs to *Musa* genre, as a monocot. Banana is the most important crop in Canary Islands, which are the most important producers of bananas in Europe. It is important to highlight that fibers are obtained from the pseudostems of the plant once the fruit has been harvested, and that each plant only bears fruit once; this is one of the main benefits of banana fibers in comparison with other natural fibers, as this one is obtained from an agricultural residue.

The use of natural fibers as reinforcement of polymeric parts has been widely studied, specially focusing on injection molding technology. In fact, around 21,000 tons of natural fibers were used in 2003 in the European industry; the most relevant fibers for the industrial production of plastic composites are flax, sisal and hemp. Natural fibers are less harmful to humans, machinery and the environment, thus being realistic alternatives to glass fiber [2].

Some studies have also been carried out for the compression molding technology, some with long fibers [3,4] and others with woven fibers [5,6,7], both for thermoset and thermoplastic polymers. These studies show that specific mechanical properties of natural fibers composites are similar to those reinforced with glass fiber, although mechanical properties under humid conditions show an important decrease for the natural fiber composites, due to their moisture absorption. These studies mainly focus on the production of non-structural parts for the automotive sector [7].

It is known that mechanical properties of composites strongly depend on the orientation of the fibers, getting better properties when the fiber is woven and placed in the composite in an appropriate orientation. The BANTEX project (MAT2013-47393-C2-1-R) is aimed at obtaining a composite material made of woven banana fibers. Woven fabrics have the advantage of enabling the orientation of the fibers, allowing control of the density of the fabric and its mechanical properties. Nonwovens provide multiaxial orientation but lower and non-predictable mechanical properties. There is wide bibliography about the production of composite materials with natural fibers (flax, hemp, jute) in woven and non-woven fabrics [8,9,10,11]; however, no references have been found in the use of banana fibers to produce them. Furthermore, the use of banana fibers to produce yarns for technical textile products has not been reported.

Banana fibers are made of cellulose (43.6%), hemicellulose (14%), lignin (11%) and other substances (such as pectin, wax, 31.4%) [12]. Chemical methods for fiber extraction are usually performed with NaOH, although other chemicals are also used (KMnO_4_, benzoyl chloride, stearic acid, among others); these processes may cause environmental problems due to the need for treating the residues produced. Mechanical means are not able to remove the non-cellulosic constituents (lignin). An alternative is the use of biological processes, such as the immersed [13] or solid state [14] fermentations. Enzymatic means are considered more environmentally friendly, and also avoid the fibers breakage, while altering the properties of the cellulosic fibers [15]. There are different parameters which affect the enzyme choice, such as the type of substrate, composition, size, lignin content, *etc.* [15]. Previous studies show Pectinase [16] and Xylanase [17] as the most suitable ones for fiber extraction. Enzymatic treatments have been applied to hemp, flax or pineapple for fiber refining. Celullases are used to remove fibrils from the surface and increase the smoothness of the fiber, although this treatment can also damage the fibers and reduce their mechanical properties. Pectinases are used in the textile industry for retting and degumming fiber crops, as they are capable of breaking down complex molecules of plant tissues into simpler ones, such as galacturonic acid [18]; on the other hand, endoglucanases only act on amorphous celluloses.

Hemicellulases are able to reduce water absorption by pentosan hydrolysis; xylanase and mannanase are used to dissolve hemicellulose (mainly xylan and glucomannan, respectively) [19].

Tests have been performed using a cocktail of two different enzymes made of pectinase and hemicellulase. In this research, different formulations of the enzymatic treatment have been applied to the banana fibers in order to determine the optimal conditions (time, temperature, enzymes content, bath renewal, fiber/enzymes ratio, *etc.*) for the refining process, in order to obtain a banana textile grade fiber. Fibers have been characterized prior to and after the enzymatic treatment, in terms of length, diameter and thermal stability. The spinning process at the lab scale has taken place, resulting in the production of a yarn with enough quality to be woven and produce a technical textile suitable for composite reinforcement.

## 2. Results

### 2.1. Fiber Treatments

A design of experiments (DoE) was made for the enzymatic treatment of the fibers, taking into account the composition and characteristics of banana fibers. Two enzymatic formulations were applied to the fibers: Biopectinase M01 (made of pectinase and hemicellulase) and Biopectinase K (made of poligalacturonase). The chosen factors are treatment time and concentration of enzymes related to fiber weight (r.f.w.). Fiber to bath volume ratio was set to 1:40 for scalability purposes, while temperature and pH were fixed according to datasheets for the selected enzymes: pH = 4.5 and T = 45 °C. The design of experiments was done using Design Expert 9.0^®^ (Stat-Ease, Inc., Minneapolis, MN, USA), varying the treatment time from 1 to 8 h and the enzyme concentration from 1% to 5% r.f.w.

The DoE was applied using each enzyme formulation with varying the treatment time and the concentration of enzymes (r.f.w.), at the same pH and temperature. As Biopectinase M01 is less active, the amount of enzymes used is 44% higher than for Biopectinase K.

**Scheme 1:**
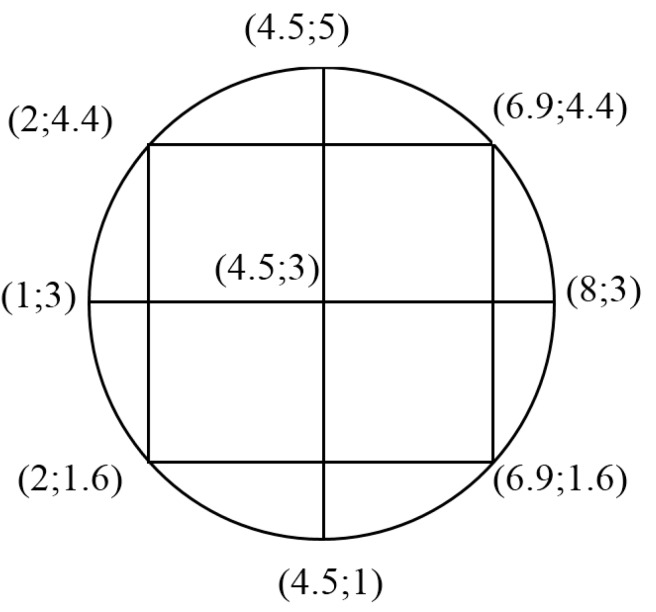
DoE for each enzyme.

The DoE is a Box–Hunter Central composite, with rotation factor of 1,41. The design is (*X,Y*)–(time, concentration). Scheme 1 shows the DoE, while Table 1 lists all performed experiments.

**Figure 1:**
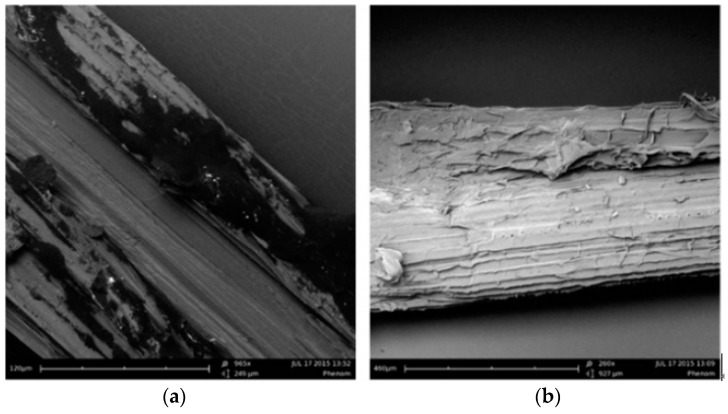
SEM pictures of banana fibers: (**a**) fibers treated with Biopectinase M01; (**b**) fibers treated with Biopectinase K.

Microscopic observations were carried out to determine the extent of the removal of undesired compounds (hemicellulose, pectin) from the fibers and their surface structure. Biopectinase K tests showed better results, even though not enough cleanliness was achieved (Figure 1).

The following step was the preparation of a new DoE, consisting of 6 experiments performed varying the time, enzyme (Biopectinase K) concentration, contact surface and fiber to bath ratio, in order to determine the limit conditions (summarized in Table 2). Temperature was kept at 45 °C, pH at 4.5 and stirring at 200 rpm.

Stability of the Biopectinase K enzyme in the operating conditions was used to determine the maximum reaction time, determining the optimum point for the bath renewal. Enzymatic activity was determined through the concentration of galacturonic acid in the bath. The poligalacturonase activity was measured in samples of 5 cm long banana fiber treated for 24 h, with a Biopectinase K concentration of 50%, at the conditions indicated above. Enzyme activity was measured each 30 min, for the first 4 h, every hour for the following 4 h and finally at the end of the test (24 h).

Taking into account results obtained from the activity test, a final DoE was performed, adapting the treatment time and enzyme concentration. Treatment was performed using Biopectinase K at 45 °C and pH 4.5, varying the treatment time from 3 to 9 h and the enzyme concentration, related to fiber weight, from 25% to 100%. From previous experiments, it was determined that an enzymatic bath should be renewed every 3 h.

**Scheme 2:**
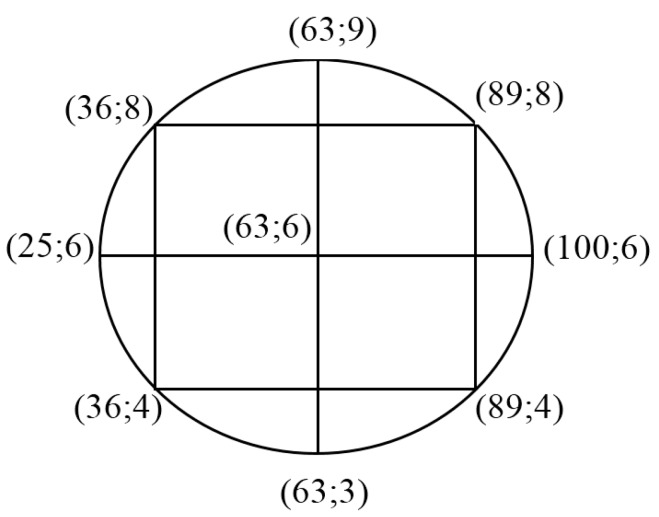
DoE for each enzyme.

The design is a Box–Hunter Central composite type with a rotation factor of 1.41. The design is (*X,Y*)–(time, concentration). Scheme 2 shows the DoE and Table 3 lists all performed experiments.

Results of mechanical testing for the above mentioned fiber together with microscopic observations have led to the reduction of experiments in two enzymatic treatments: Biopectinase K enzyme at 25%, and 100% concentration r.f.w. for 6 h, with bath renewal after 3 h of treatment. These treatments will be referred to as Treatment 1 and Treatment 2, respectively. Fibers after Treatment 2 were carried to a pilot scale yarn production plant for spinning tests.

### 2.2. Fiber Characterization

#### 2.2.1. SEM Microscopy

Results of the first DoE (conditions shown in Table 1) were analyzed by SEM microscopy, as fibers have not shown good enough macroscopic quality to carry out the characterization tests. Images obtained at the same magnification show that fiber depletion does not occur, thus indicating that performed treatments were not enough to reach the target quality for a textile fiber degree. However, it can also be concluded that treatment with Biopectinase K seems to provide better results than Biopectinase M01, as the fibers appear cleaner.

**Figure 2:**
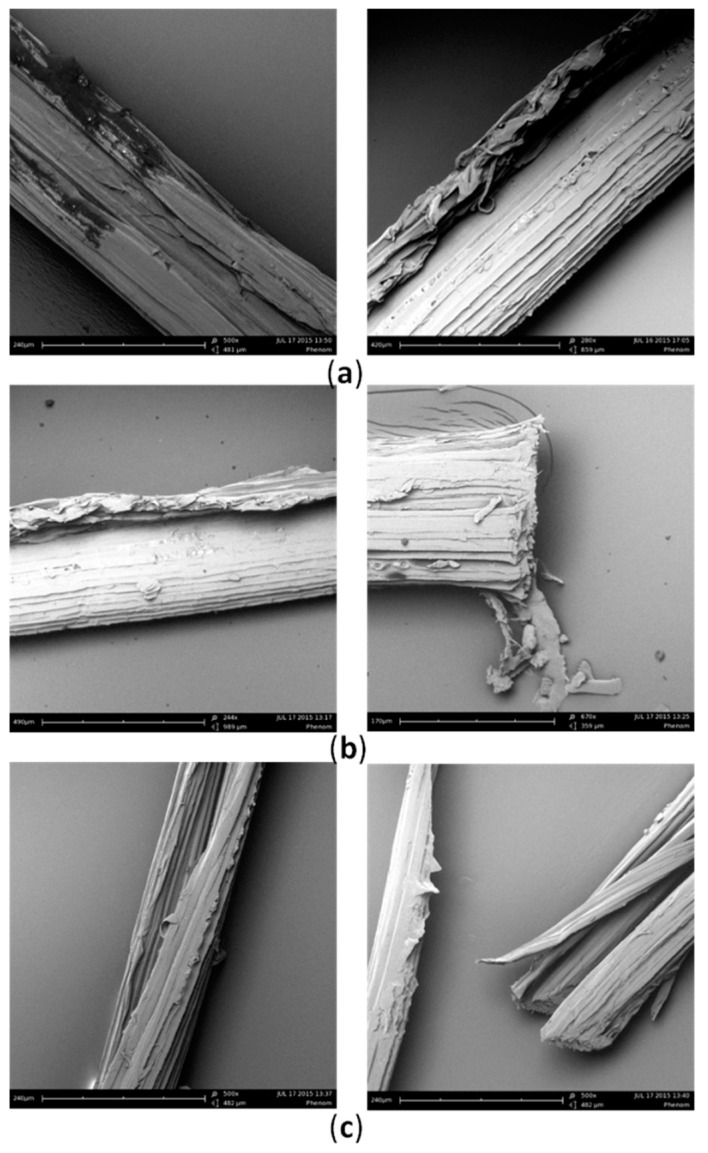
SEM pictures of banana fibers: (**a**) untreated fibers; (**b**) fibers after Treatment 1; (**c**) fibers after Treatment 2.

Observations carried out for selected treatments (25% and 100% r.f.w. for 6 h: Treatments 1 and 2) as well as for virgin fibers, show that treated fibers are cleaner than virgin ones. Furthermore, as observed in Figure 2, fibers from Treatment 2 are the thinner ones, showing microfibrillation, due to the removal of hemicellulose and pectin, which act as bonding agents between the microfibrils that form a fiber; these bonding substances can be observed in untreated fiber images.

#### 2.2.2. Optical Microscopy

**Figure 3:**
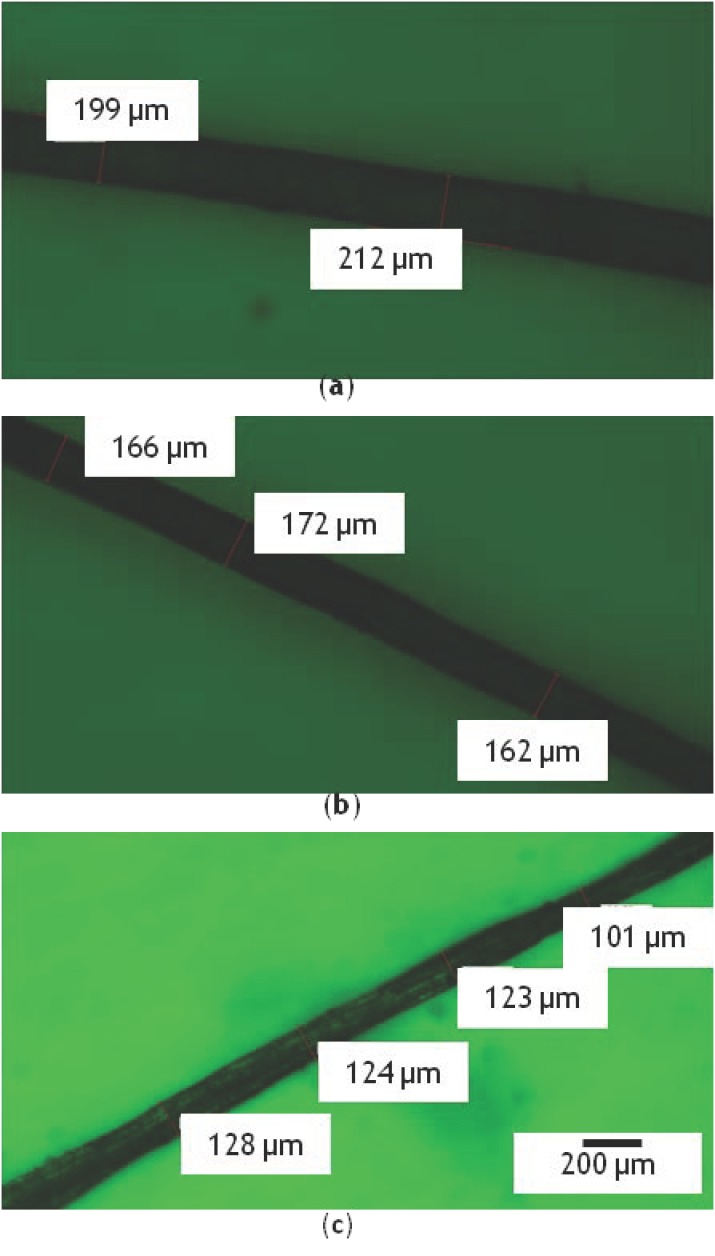
Optical micrographs of virgin banana fibers and their diameters: (**a**) untreated fibers; (**b**) fibers after Treatment 1; (**c**) fibers after Treatment 2.

Banana fibers were observed at different magnifications, under normal and polarized light. Ten individual fibers were measured. Virgin banana fiber had an average diameter of about 200 μm, while treated fibers show smaller average diameters: 160 µm for fiber after Treatment 1 and 114 µm for Treatment 2 fibers. Figure 3 shows an image of each type of fiber at the same magnification, where the reduction of the fiber diameter can be clearly observed. Diameters of micro-fibrils measure approx. 23 µm for virgin fibers and 16 μm for treated ones.

#### 2.2.3. Mechanical Properties of Banana Fiber

Up to 10 samples of fiber were tested. Results show a tensile strength of 42.8 ± 6.5 cN/tex for virgin banana fibers. As can be observed in Table 4, the tensile strength of fibers with Treatment 1 is virtually unchanged, while a reduction of 14% is observed for fiber with Treatment 2.

Table 4 shows the results obtained for mechanical testing of fibers after the selected treatments; however, as explained in Section 2.1, mechanical tests were also performed for fibers treated according to Table 3. Results obtained for 6 h treated fibers are summarized in Table 5.

#### 2.2.4. Thermal Stability

**Figure 4:**
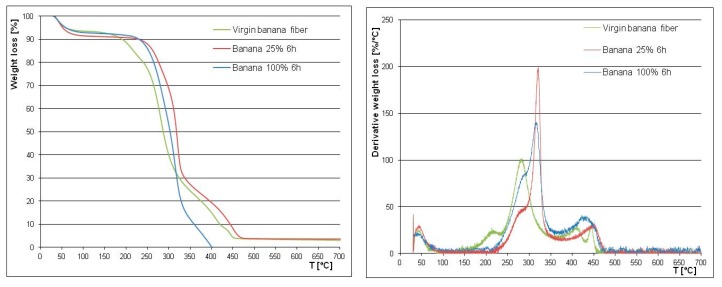
TG and DTG curves.

Table 6 shows the average values obtained in thermal tests using thermogravimetric analysis (TGA). It is clearly observed how the thermal stability of the fibers is increased significantly due to the enzymatic treatment. The left limit temperature refers to the temperature at which weight loss starts (apart from humidity removal). Onset temperature is obtained from the intersection between the protractions of the flat part of the curve before the left limit temperature and the most important weight loss section of the curve; as it is needed from graphical calculations, it may be an inexact parameter, but it has been calculated for comparison purposes with bibliography data. Peak temperature corresponds to the maximal degradation rate, and it is obtained from the weight loss derivative curve. Results can be more clearly observed in Figure 4.

Isothermal tests (carried out at 220 °C) show higher weight loss for untreated fiber (39.7%); an important decrease in this parameter was observed for treated fibers: 25.2% and 15.2% for fibers after Treatments 1 and 2, respectively. This parameter confirms the higher thermal stability achieved from the enzymatic treatments due to the removal of less thermally stable substances (mainly hemicelluloses and pectin). This temperature was chosen as it is a usual temperature in thermoplastic parts processing.

### 2.3. Processability: Spinning Process

#### 2.3.1. Opening

This stage was performed in a lab-scale Shirley opener for short fiber. As banana fiber shows high stiffness (around 360 cN/tex) compared to other fibers such as flax or hemp (150 cN/tex) or cotton (80 cN/tex), higher amounts of fiber are collected on the tray, even when varying the blades and working rates. However, the processed fiber is more open and its processing is improved in the combing flats. Some mechanical adjustments could be done to improve this first step yielding.

#### 2.3.2. Revolving Flat Card

**Figure 5:**
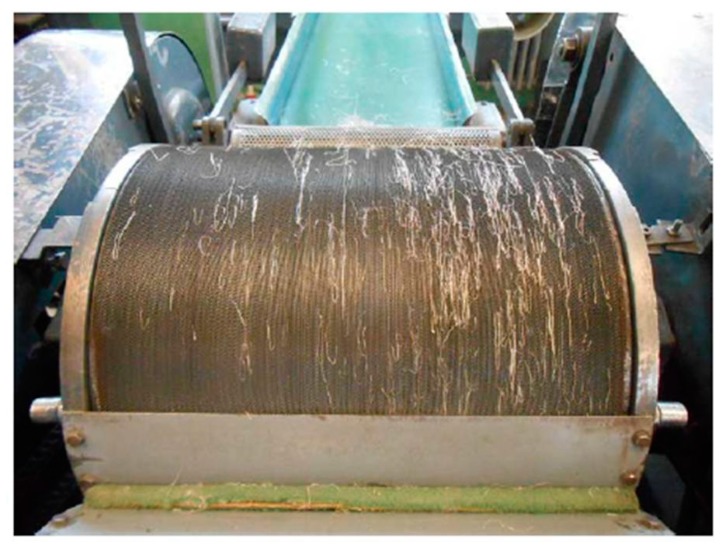
Adherence of the fibers to the drum during the carding process.

This part of the work was conducted on a Platt card with rigid cards, at high levels of humidity (17 g water per dry air kg). First, results showed broken fibers with low interfibrillar cohesion; fibers are adhered to the drum, the flats ant the comber noils, as shown in Figure 5. Furthermore, an important amount of scrap is produced during the processing, which reflects the difficulty of spinning this fiber.

**Figure 6:**
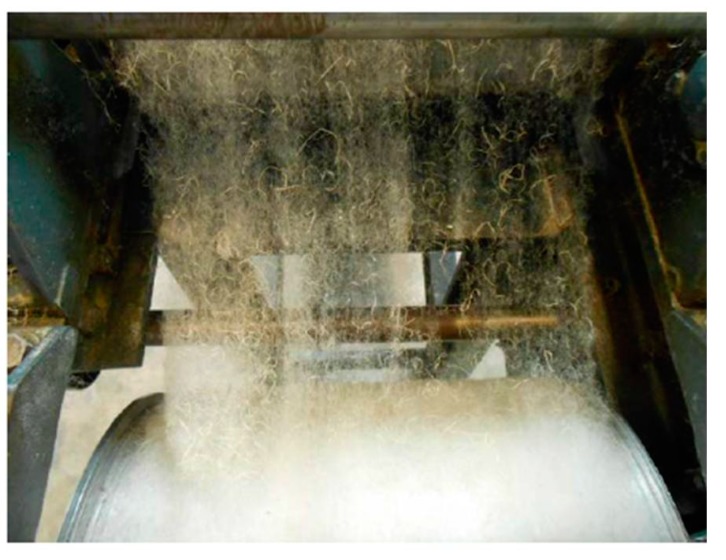
Veil cohesion for 50/50 blend of banana and cotton fibers.

To try to improve the efficiency of the process, banana fibers were mixed with cotton at different ratios: 50/50 and 70/30 (banana/cotton). Cotton is acting in this yarn as a supporting fiber. Fibers, after passing through the Shirley opener, were blended together manually and then processed in the revolving flat card. It can still be observed that part of the fibers adhere to the drum and the cards although in a smaller proportion, getting a veil with better interfibrillar adhesion (Figure 6) and less scraps produced.

As expected, when increasing the ratio of banana fiber up to 70%, the behavior in the carding process is more similar to this for 100% banana fiber, with more damaged fibers and increased adherence to the drum and the card flats (relating to the 50/50 blend). An optimization in the machinery could lead to an improvement in the carding stage for banana fibers, achieving better processability.

**Figure 7:**
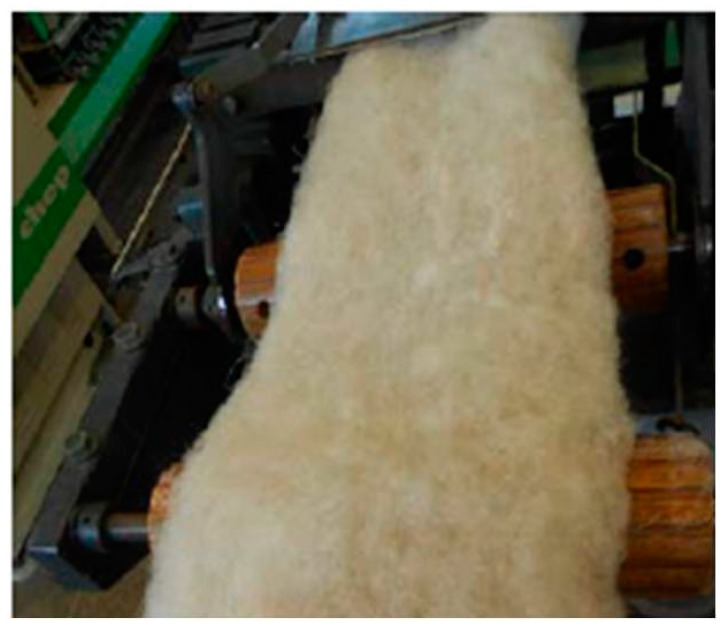
Veil of banana-wool blend 70/30.

Further tests were conducted using polyester conventional fibers as support fiber, at a 50/50 ratio. Results were similar to those obtained with the cotton blend. Finally, wool was also used, in order to increase the amount of banana fiber in the mix, taking advantage of the natural curling in wool as a cohesion element; blends were made with 50% and 70% of banana fibers. The procedure was identical to the one followed for cotton. This blend can be processed in a conventional rubin device under industrial conditions, although at a lower production rate than conventional cotton. Figure 7 shows the result of the carding process for 70% banana/30% wool blends.

Table 7 shows a summary of the conditions followed for the drawing frame process.

**Figure 8:**
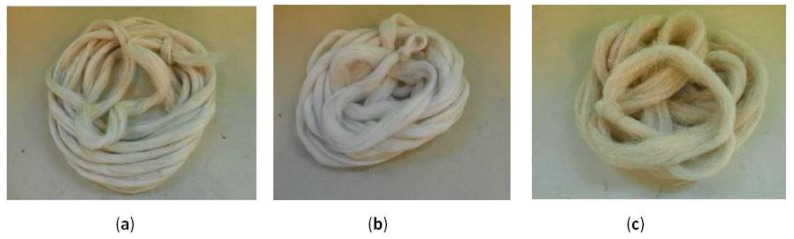
Drawing sliver of the banana fiber blended with: (**a**) cotton; (**b**) polyester; (**c**) wool.

**Figure 9:**
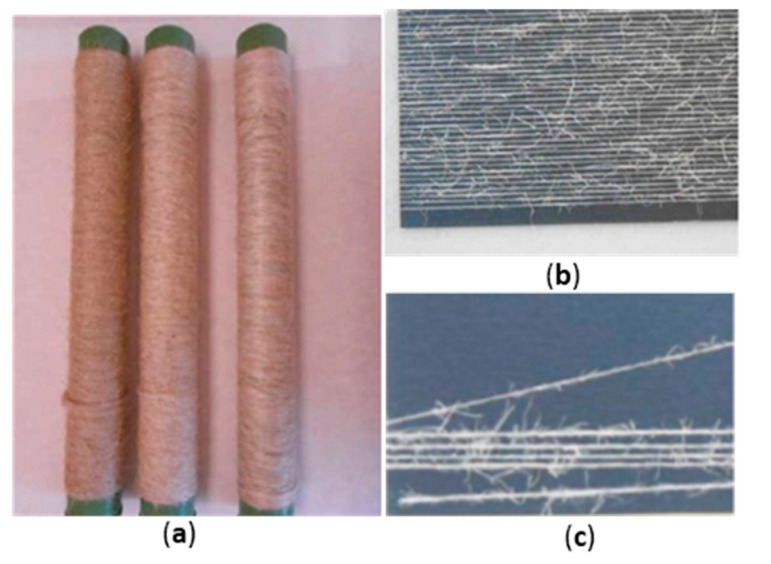
Pictures showing: (**a**) rovings obtained with the three blends; (**b**) banana/wool yarn; (**c**) 2 ply banana/wool yarn.

Figure 8 shows images of the drawing sliver obtained for different blends. They were spun after to obtain the rovings and then twisted to obtain the yarns and two ply yarns (Figure 9).

As a result of the experiments performed, it is observed that banana fibers can be spun to get yarn, although the machinery should be adapted to the high stiffness of these fibers. Better results are obtained when blending banana fibers with other softer fibers; at this stage, the most suitable blend to be obtained at industrial scale would be this with wool.

### 2.4. Yarn Characterization

Table 8 shows average results obtained for banana and banana/PP yarn. This yarn was made with the blends made of 70% banana fibers and 30% wool. The yarn containing PP fiber was made twisting a banana yarn with PP yarn (330 dtex), at 90 laps/m, in an S sense. Tests on a conventional flax/PP yarn were also conducted for comparison. It is observed that banana/PP yarn has higher tenacity than flax/PP yarn and also that elongation at the break is higher for the banana yarn than for flax/PP yarn.

## 3. Discussion

### 3.1. Microscopy

As explained in the Results section, fibers after the enzymatic treatment appear cleaner and defibrillated, where this effect is more observed at higher enzymes concentration. In addition, it was proven that Biopectinase K provided better cleanliness than Biopectinase M01. Polygalacturonase (Biopectinase K) belongs to the pectinases family, while Biopectinase M01 consists in a blend of hemicellulases and pectinase. Pectin hydrolysis has been found useful for banana fiber cleaning, at 45 °C for 90 min [16]; however, defibrillation does not occur and fiber diameter does not seem to be affected. The Fangamyx enzyme was used to treat abaca fibers [20], resulting in the presence of defibrillation and a reduction in diameter, although less significant (from 145 to 130 µm) than the one observed in this work for banana fibers. Hemp has also been treated using enzymes (SIHA-Panzym^®^ DF, pectinase) with the final aim of producing composites, resulting in a decrease of 42% in the cross section [21].

Cotton fibers show a diameter of 15.6 µm, and it is well known that they are the purest fibers among the natural ones, being mainly constituted of cellulose [22]; fibrils measured for treated banana fibers show a diameter around 16 μm, while virgin ones are around 23 µm; this shows also the cleanliness degree achieved after the enzymatic process with the removal of pectin and hemicellulose.

### 3.2. Mechanical Properties of Banana Fiber

Virgin banana fibers (42.8 cN/tex) show higher tenacity than hemp (23.6 cN/tex [23]), jute (23.9–27.6 cN/tex [24]) or cotton (24–36 cN/tex [25]), while having a lower tenacity than flax (59.9 cN/tex [26]), sisal (57.2 cN/tex [27]) or rayon (56 cN/tex [26]). Treated banana fibers show better results than hemp, jute or cotton, while showing reduced compared to untreated flax or hemp. Fibers obtained from Colombian plantains exhibit a tensile strength of 47 cN/tex [28].

From Table 4 and Table 5, it can be observed that enzyme concentrations over 25% r.f.w. result in lower mechanical properties due to the most intensive cleaning process. As explained in Section 2.2.1, the fiber surface appears cleaner with the highest concentration of enzymes, leading to the defibrillation of the fibers into the cellulosic microfibrils, a fact which explains the stiffness decrease. An increase in the mechanical properties of flax has been reported after fiber retting [29]. The authors explain this by the lower diameter and weight of the fibers once they have been retted (from 47.1 to 72.8 cN/tex). Other research also shows a decrease in the mechanical properties due to their treatment and processing; hemp tenacity is reduced from 23.6 to 8.5 cN/tex after being immersed in an 18% NaOH solution for 30 min at room temperature [23], while varying from 29.8 to 24.9 cN/tex after mercerization with NaOH 22% solution for 60 min [30].

### 3.3. Thermal Stability

Comparing data obtained from TGA tests summarized in Table 6 with those obtained for major constituents in the fiber (cellulose, hemicellulose and lignin), it is possible to infer the extent of hemicellulose and lignin removal because of the enzymatic treatment.

Hemicellulose degradation temperatures are found between 220 °C and 278 °C (pectin also degrades at this temperature range [31]); it was found that weight loss at this stage is significantly decreased due to the enzymatic treatment (from 30.6% to 21.3% and 12.2% for virgin fibers, 25% and 100% 6 h treated fibers). From these results, it can be inferred that part of the hemicelluloses have been degraded during the enzymatic treatment. Following the degradation step, which takes place between 278 °C and 360 °C, corresponding to cellulose degradation; the weight loss in this part of the curve increases with the enzymatic treatment, that is, the amount of cellulose is higher in fibers which have been treated. This corresponds to the previous observation of the lower content in hemicelluloses, and with the higher fineness of the fibers, as hemicelluloses act as a bonding agent among the cellulosic fibrils. Peak temperature for all fibers correspond to the degradation of cellulose, and degradation rates go from 1.0 wt%/°C for virgin fibers to 2.0 wt%/°C for treated ones (pure cellulose has a degradation rate of 2.8 wt%/°C [32]). The difference between the pure cellulose and the banana fibers may be due to the presence of non-cellulosic compounds, such as lignin, in the fibers. Higher temperatures lead to lower decomposition rates, related to lignin degradation; lignin degrades from the beginning of the test until 900 °C [32]; degradation of fibers until 900 °C show higher residues for virgin fiber; this is also observed in ash content at the end of the test (1100 °C), being 3.8% for virgin and 25% treated fibers; and only 1% for 100% treated ones. This ash could be due to the presence of inorganic compounds in banana fibers, non-affected by enzymatic treatments.

An increase in the thermal stability due to the enzymatic treatment is clearly shown by the three temperatures shown in Table 6; other authors have also reported increases from 10 to 20 °C due to the fiber treatment, either chemical or enzymatic [20,33].

### 3.4. Yarn Characterization

Other authors show a tenacity for flax yarn of 14.8 cN/tex [34] and 16 cN/tex for bamboo yarn [35]. Banana/PP yarns show around 46% higher tenacity than flax/PP yarn, also with a lower coefficient of variation; this means that the banana/PP samples are more homogeneous. Banana fiber has lower tenacity (5.5 cN/tex) than previously reported yarn, although Banana/PP yarn is tougher than for the flax/PP one. The toughening is achieved by the type of yarn Core Spun and using the optimum torsion laps between banana/wool and PP yarn.

The spinning process has been slightly modified from the conventional process, especially for card clothing and the machine parameters like speed, distance and position of the cylinders. The 3 stage drawing frame is necessary to achieve a good mix of banana and wool fibers, and to obtain regular good yarn tenacity.

## 4. Materials and Methods

### 4.1. Materials

Banana fibers were produced at the Universidad de Las Palmas de Gran Canaria (ULPGC) facilities (Las Palmas de Gran Canaria, Spain), by means of a patented system by ULPGC. This prototype equipment enables extracting fiber from the leaves by scraping it in two stages: linear scrapping and rotational scrapping (potential production rate 90 kg/h). Afterwards, the long fiber (0.5–1 m) is chopped in a developed machine, in the context of this research work, for cutting the fiber with a high level of uniformity. The fiber was cut into small lengths according to the requirements of the enzymatic treatment (45 mm). Enzymatic coctels used were obtained from BIOCON (Les Franqueses del Vallès, Spain), Biopectinase M01 and Biopectinase K, containing pectinase and hemicellulase.

Conventional fibers were obtained from Innotex Center CTF (Terrassa, Spain). Cotton fibers used in the blend with banana fibers were 28 mm long and had 3.8 in micronaire. PP fibers were cotton-like cut, 38 mm length and 330 dtex fineness. Polyester fibers were cotton-like cut, conventional mat, 38 mm long, 1.5 dtex. Finally, wool fibers had 65 mm length and 23 µm fineness.

### 4.2. Methods

#### 4.2.1. Fiber Teatments

A design of experiments (DoE) was made for the enzymatic treatment of the fibers, taking into account the composition and characteristics of banana fibers. Two enzymatic formulations were applied to the fibers: Biopectinase M01 (made of pectinase and hemicellulase) and biopectinase K (made of poligalacturonase). The variable parameters chosen are treatment time and concentration of enzymes related to fiber weight (r.f.w.). Fiber to bath volume ratio was settled to 1:40 for scalability purposes, while temperature and pH were fixed according to datasheets for the selected enzymes: pH = 4.5 and T = 45 °C.

#### 4.2.2. Fiber Characterization

Fibers were characterized by means of thermogravimetric and isothermal analysis, microscopy, and fineness. Thermogravimetric analyses (TGA) were run to determine potential thermal degradation of fiber; a Mettler Toledo TGA/DSC1 analyzer (Mettler Toledo, Toledo, OH, USA) was used at a heating rate of 5 °C/min in air atmosphere (10 mL/min). Isothermal studies were also carried out by keeping the fibers at 220 °C for 150 min, in air atmosphere with 10 ml/min of air flow. Three replicas were performed for these tests. Microscopic observations of fibers were carried out in an Olympus BX51 optical microscope (Olympus, Tokyo, Japan), at different magnifications and under polarized light; 10 samples were measured for each type of fiber (5 measures in each fiber). SEM microscopy was made in a Tabletop Phenom device (Phenomworld, Eindhoven, The Netherlands) in the fiber direction to observe its surface. Mechanical tests of fibers were performed according to UNE EN-ISO standard 5079:1996, in a constant elongation gradient dynamometer from Instron (mod. 4501) (Norwood, NJ, USA); samples were conditioned at 20 °C ± 2 °C and 65% ± 4% humidity for 24 h. These conditions were also kept during testing. 10 samples were tested, at 10 mm/min.

#### 4.2.3. Spinning Process

Once fibers were treated by enzymatic means and characterized, fibers were introduced into a lab-scale spinning plant. A Shirley opener for short fiber (SDL Atlas, Rock Hill, SC, USA) was used before introducing the fibers into the carding flats. The absolute humidity during fiber processing was 17 g/kg dried air, as previous experiences have shown that stiff fibers have better processability when working at high humidity rates. Due to the difficulty of banana fibers spinning (because of their high stiffness), they were mixed with cotton, polyester and wool. Once the wick was produced, the following step was carried out in an industrial twist roving Electro-Jet (Electro-Jet S.A., Gurb, Spain). The spinning process took place in a continuous ring spinning machine, from Pinter (Pinter S.A., Santpedor, Spain). The yarn was then twisted in a ring twister from Galan Textile Machinery (Terrassa, Spain); the filaments were twisted at 400 laps/m, in an S sense; and the spindles ratio was 2000 laps/min with a production of 5 m/min.

#### 4.2.4. Yarn Characterization

Produced yarns were characterized to determine their title and stiffness. Mechanical testing was carried out following the standard procedure scheduled. UNE-EN ISO 2062-2010 (Method A) was followed, using a constant elongation gradient dynamometer INSTRON (Class 0.5) (Instron, Norwood, NJ, USA). Samples were conditioned for 24 h at 20 °C ± 2 °C y 65% ± 4% r.h. Specimens were 250 mm long, tensile speed was 250 mm/min and a pretension of 0.5 cN/tex was applied. 20 replicas were tested. Title was determined following the standard method of UNE-EN ISO 2060:1996.

## 5. Conclusions

- The enzymatic treatment has proven to be useful for banana fiber treatment, achieving an improvement in terms of cleanliness and fibrillation.
- The most effective enzyme for banana fiber treatment is poligalacturonase (Biopectinase K), showing a high specific activity and being specific for substrates not damaging the cellulosic structure of fibers.
- Long duration treatments (24 h, 48 h and 7 days) did not provide good results, due to enzyme deactivation. 6 h was optimal to obtain a textile grade banana fiber.
- Optimal conditions for banana fiber enzymatic treatment are: 100% Biopectinase K, 6 h; 45 °C, pH = 4.5, with bath renewal after 3 h.
- Stability studies have demonstrated that over 80% of its activity takes place in the first 3 h; afterwards, the enzyme activity decreases reaching 12% 24 h later.
- Enzymatic treatments improve the thermal stability of fibers by the removal of pectin and hemicellulose, while producing a slight decrease in mechanical properties, probably due to defibrillation found under SEM observations.
- Banana fiber can be spun to produce yarns, mixed or not mixed with other fibers, while the most suitable for industrial scale-up without major equipment changes would be the blend of banana fiber and wool.
- Banana/PP yarn shows higher tenacity than flax/PP yarn and is more homogeneous.

## Figures and Tables

**Table 1:** Treatment conditions (for each enzyme) for the first DoE.

Test Number	Time (h)	Enzyme Concentration (% r.f.w)
1	6.98	4.410
2	4.50	4.996
3	8.01	2.995
4	4.50	2.995
5	4.50	0.994
6	4.50	2.995
7	2.02	4.410
8	4.50	2.995
9	0.99	2.995
10	2.02	1.580
11	4.50	2.995
12	4.50	2.995
13	6.98	1.580

**Table 2:** Tests to determine limit conditions for Biopectinase K.

Test Number	Time (h)	Enzyme Concentration (% r.f.w)	Fiber/Bath Ratio	Fiber Length (cm)
1	24	10	1/40	Long ^1^
2	24	10	1/40	5
3	24	20	1/40	5
4	48	20	1/40	5
5	48	20	1/80	5
6	168	20	1/40	5

^1^ This is a fiber with the length obtained from the plant after the mechanical extraction procedure; in this case, around 0.6 m.

**Table 3:** Treatment conditions for Biopectinase K treatments in the last DoE.

Test Number	Time (h)	Enzyme Concentration (% r.f.w)
1	6.00	100.00
2	3.88	89.00
3	3.00	62.50
4	3.88	35.91
5	6.00	25.00
6	8.12	35.91
7	9.00	62.50
8	8.12	89.09
9	6.00	62.50
10	6.00	62.50
11	6.00	62.50
12	6.00	62.50
13	6.00	62.50
14	6.00	35.91
15	6.00	89.00

**Table 4:** Banana fiber tenacity.

Fiber	Tenacity (cN/tex)	Standard Deviation
Untreated	42.8	6.5
Treatment 1	42.0	5.4
Treatment 2	36.8	6.3

**Table 5:** Tenacity of the different fibers treated in the last DoE.

Fiber	Tenacity (cN/tex)	Standard Deviation
62.50% 6 h	28.6	4.7
89.09% 6 h	35.2	6.4
35.90% 6 h	30.7	6.2

**Table 6:** Degradation temperatures (in °C) of virgin and treated banana fibers.

Fiber	Left Limit	Onset	Peak	Humidity (%)	Ashes (%)
Untreated	180	245	280	6.5	3.8
Treatment 1	220	300	321	8.6	2.9
Treatment 2	245	290	316	6.1	1.0

**Table 7:** Conditions for drawing frame the blends with banana fibers.

Parameter	1st Stage	2nd Stage	3rd Stage
Feed slivers (number)	Nappa	4	2.65
Joining slivers (number)	–	4	6
Previous stretching (rate)	1.3	1.3	1.3
Total stretching (rate)	9	6	3.5
Output title (g/m)	4	2.65	4.6
Gauge of the rubin (mm)			
Wool blend (%)	50/58/45	50/58/45	50/58/45
Polyester blend (%)	48/45/42	48/45/42	48/45/42
Cotton blend (%)	38/35/32	38/35/32	38/35/32
Pressure (method)	Springs	Springs	Springs
Output rate (mm/min)	20	20	20

**Table 8:** Mechanical properties of yarn.

Property	Banana Yarn	Banana/PP Yarn	Flax/PP Yarn
Title of the yarn (tex)	166	166	166
Strength of breaking load (cN)	910	1910	1310
Coefficient of variation (%)	11.2	4.9	12.4
Tenacity (CN/tex)	5.5	11.5	7.9
Elongation at break (%)	13.0	19.7	4.5
Coefficient of variation (%)	21.3	6.4	26.5

## Abbreviations

The following abbreviations are used in this manuscript:

r.f.w.	related to fiber weight;
TGA	Thermogravimetric Analysis;
DoE	Design of Experiments;
SEM	Scanning Electron Microscope.
